# Syrian Hamsters Model Does Not Reflect Human-like Disease after Aerosol Exposure to Encephalitic Alphaviruses

**DOI:** 10.3390/mps7030042

**Published:** 2024-05-15

**Authors:** Christina L. Gardner, Rebecca A. Erwin-Cohen, Bridget S. Lewis, Russell R. Bakken, Shelley P. Honnold, Pamela J. Glass, Crystal W. Burke

**Affiliations:** 1 Virology Division, U.S. Army Medical Research Institute of Infectious Diseases, Frederick, MD 21702, USA; christina.l.gardner8.ctr@health.mil (C.L.G.); rebecca.erwin-cohen@nih.gov (R.A.E.-C.); russell.r.bakken.civ@health.mil (R.R.B.); pamela.j.glass.civ@health.mil (P.J.G.); 2 Pathology Division, U.S. Army Medical Research Institute of Infectious Diseases, Frederick, MD 21702, USA; bridgetslewis757@gmail.com (B.S.L.); shelleyhonnold@gmail.com (S.P.H.); 3 Risk Management Office, U.S. Army Medical Research Institute of Infectious Diseases, Frederick, MD 21702, USA

**Keywords:** alphavirus, hamsters, aerosol, animal model, Venezuelan equine encephalitis, eastern equine encephalitis, western equine encephalitis

## Abstract

Venezuelan (VEE), eastern (EEE), and western (WEE) equine encephalitis viruses are encephalitic New World alphaviruses that cause periodic epizootic and epidemic outbreaks in horses and humans that may cause severe morbidity and mortality. Currently there are no FDA-licensed vaccines or effective antiviral therapies. Each year, there are a limited number of human cases of encephalitic alphaviruses; thus, licensure of a vaccine or therapeutic would require approval under the FDA animal rule. Approval under the FDA animal rule requires the disease observed in the animal model to recapitulate what is observed in humans. Currently, initial testing of vaccines and therapeutics is performed in the mouse model. Unfortunately, alphavirus disease manifestations in a mouse do not faithfully recapitulate human disease; the VEEV mouse model is lethal whereas in humans VEEV is rarely lethal. In an effort to identify a more appropriate small animal model, we evaluated hamsters in an aerosol exposure model of encephalitic alphavirus infection. The pathology, lethality, and viremia observed in the infected hamsters was inconsistent with what is observed in NHP models and humans. These data suggest that hamsters are not an appropriate model for encephalitic alphaviruses to test vaccines or potential antiviral therapies.

## 1. Introduction

Alphaviruses are enveloped viruses that contain a single-stranded, positive-sense RNA genome. Alphaviruses are mosquito-vectored viruses that can cause periodic epizootic and epidemic outbreaks in horses and humans. Alphaviruses can be divided into two categories based on the geographic origin and disease characteristics of the virus. Old World alphaviruses predominately cause arthritic disease while the New World alphaviruses can cause encephalitic disease. The three members of the encephalitic New World alphaviruses are Venezuelan (VEE), eastern (EEE), and western (WEE) equine encephalitis viruses. The encephalitic alphaviruses are maintained in an enzootic cycle between the mosquito vector and the reservoir host, either rodents or birds depending on the virus [1]. During the epizootic cycle, bridging vectors infect horses and humans, with horses being an amplifying host [1]. All three viruses cause >80% mortality in horses [2] but differ significantly in their disease manifestation in humans. While all three viruses are capable of causing encephalitic disease, symptom presentation and mortality rates are very different. VEEV infection typically results in a high attack-rate of a febrile illness; however, 14–20% of symptomatic individuals develop neurological symptoms with a mortality rate of <1% [1,3,4]. Conversely, symptomatic EEEV disease occurs at a lower incidence, but the main symptoms are neurological with a 30–70% mortality in symptomatic individuals [5]. Furthermore, 35–80% of survivors will have permanent neurological impairments [1,5]. Lastly, WEEV has a low incidence rate but, similar to EEEV, mainly causes neurological symptoms in symptomatic individuals [6]. The WEEV mortality rate is 3–15% in symptomatic individuals with 15–30% of survivors having moderate neurological sequelae [1,5].

There are currently no licensed human vaccines or effective antiviral therapies for any of the encephalitic alphaviruses. As a result of the low incidence of the naturally occurring disease, FDA licensure of vaccines or antiviral therapies would occur under the FDA animal rule. The cynomolgus macaque model has been used for advanced testing of vaccines and therapeutics [7,8,9,10,11,12,13,14] because disease in this nonhuman primate (NHP) closely mimics human disease [15]. Prior to progression to the NHP model, potential vaccines and therapeutics must first be screened in a small animal model. Currently the mouse model serves as the screening model for alphavirus vaccines and therapeutics. One major limitation of the mouse model is that all three encephalitic alphaviruses are highly lethal when delivered via an aerosol route. Furthermore, disease manifestations in the mouse, especially for VEEV, do not mimic what happens in humans [16]. Hamsters have been used to study encephalitic alphaviruses since the 1960s, with most of the research using either the subcutaneous (SC) [17,18,19,20,21,22,23,24,25,26,27,28] or the intraperitoneal (IP) [12,18,22,29,30,31,32] route of infection and only a few experiments using aerosol exposures for VEEV [12,28] and WEEV [22,33]. While the SC route of infection more closely mimics natural exposure, the aerosol route of infection is of military importance.

In this study, the aerosol lethal dose 50 (LD50) of Syrian hamsters exposed to VEEV, EEEV, or WEEV was determined. Additionally, a pathology study was conducted to examine histological changes, viral tissue tropism, and level of viral antigen in the tissues.

## 2. Materials and Methods

### 2.1. Ethical Statement

This research was conducted under an IACUC approved protocol in compliance with the Animal Welfare Act, PHS Policy, and other Federal statutes and regulations relating to animals and experiments involving animals. The facility where this research was conducted is accredited by the Association for Assessment and Accreditation of Laboratory Animal Care International and adheres to principles stated in the Guide for the Care and Use of Laboratory Animals, National Research Council, 2011.

### 2.2. Animals

Syrian hamsters (*Mesocricetus auratus*) of both sexes were obtained from Harlan Scientific and randomly selected for inclusion in the LD50 estimation, pathology experiment, and vaccination study. Hamsters were 8–10 weeks old and approximately 100 g at the time of aerosol exposure. Animals were provided with rodent chow and water ad libitum and maintained on a 12 h light/dark cycle. Some hamsters were implanted with an IPTT temperature probe (Bio Medic Data Systems) to monitor temperature.

### 2.3. Viruses

Biologically derived viruses were used for this study. EEEV (FL91-4679) was passaged on BHK-21 cells two times, VEEV (Trinidad donkey) stock was received from DynPort Vaccine Company (DVC) and prepared by Commonwealth Biotechnologies Inc., and WEEV (CBA87) was passaged once on BHK-21 cells. Virus titer was determined by standard plaque assay on VERO cells [34]. All experiments were conducted in BSL-3/ABSL-3 conditions. The virus strains chosen for this study were based on the strains being used for vaccine efficacy studies occurring at the time this study was completed.

### 2.4. Aerosol

Hamsters were exposed inside whole-body chambers to aerosolized virus within a Class III biological safety cabinet operated under negative pressure. Briefly, aerosols were generated with a Collison nebulizer (BGI Inc., Waltham, MA, USA) to produce approximately 1 µm mass-median aerodynamic diameter (MADD) particles for each 10 min exposure under the control of an automated bioaerosol exposure system [35]. For the LD50 estimation, hamsters (5 groups of *n* = 10 hamsters/group) were exposed to a targeted dose range of 1 × 10^4^ to 1 × 10^−1^ PFU of aerosolized virus. For the pathology study (6 groups of *n* = 3 hamsters/group) and for the VRP vaccine study (4 groups of *n* = 10 hamsters/group), hamsters were exposed to a target dose of 100 LD50 for each virus. All animals were weighed daily and monitored for clinical signs of disease. Calculated inhaled dose was determined from the All-Glass Impinger (AGI) after each aerosol by standard plaque assay [34].

### 2.5. Pathology

Pathology studies were conducted on groups of three hamsters which were serially euthanized every 12 h beginning at 12 h post infection until 72 h post infection. Gross necropsy, histopathology, and immunohistochemistry were performed. Additionally, whole blood was collected in EDTA tubes to determine viremia using a standard plaque assay [34]. Tissues were fixed in 10% buffered formalin for a minimum of 21 days. For histopathology, tissues were trimmed and processed according to standard protocol [36]. Sections were trimmed to 5–6 µm thickness and stained with hematoxylin and eosin (H & E). A board-certified veterinary pathologist evaluated the H & E-stained tissues and immunohistochemical slides. For immunohistochemistry, a cross-reactive polyclonal rabbit antibody produced against alphaviruses was diluted and used as the primary antibody to detect viral antigens. Briefly, tissue sections were de-paraffinized and rehydrated. To increase staining intensity, an antigen retrieval procedure requiring immersion of sections in citrate buffer was used and endogenous peroxidases were blocked by incubation with hydrogen peroxide. Tissue sections were incubated with the appropriate dilution of primary rabbit antibody followed by the secondary antibody, horseradish peroxidase-labeled polymer conjugated to goat anti-rabbit immunoglobulin. Staining was completed by adding 3,3-diaminobenzidine (DAB). Tissues were counterstained with hematoxylin, dehydrated, cleared, and covered. Results for each animal were documented on individual pathology reports and immunohistochemical findings summarized for each virus.

### 2.6. Statistics

The median lethal dose (LD50) was determined by employing a Bayesian probit analysis performed for each virus to estimate the effective dose–response curve. Prior distributions for the slope and intercept parameters for each probit regression were chosen to be weakly informative Cauchy distributions with center 0 and scale 10. Using samples from the posterior distributions of the slope and intercept parameters from the probit analysis, the median and 95% credible intervals of the range of dose responses are estimated. A two-way ANOVA (GraphPad version 9.4.0) was used to determine statistical significance of the mock animals compared to infected animals at each time point for temperature.

## 3. Results

### 3.1. Lethal Dose 50 (LD50) of Hamsters Aerosol Exposed to Encephalitic Alphaviruses

**Figure 1:**
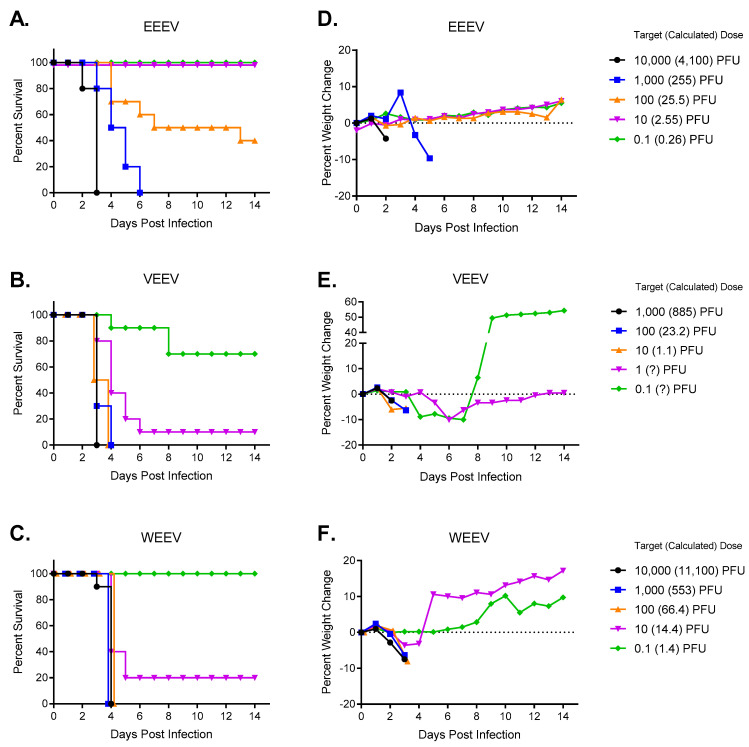
Determination of LD50 in hamsters after aerosol exposure to encephalitic alphaviruses. Eight- to ten-week-old hamsters of both sexes (*n* = 10 per group) were exposed to five target doses of viruses ranging from 0.1 PFU to 10,000 PFU of aerosolized EEEV (**A**,**D**), VEEV (**B**,**E**), and WEEV (**C**,**F**). Hamsters were monitored daily for morbidity and mortality: survival curves (**A**–**C**) and weight (**D**–**F**). The calculated inhaled dose was determined by the concentration of virus in the AGI after each aerosol. The LD50 was calculated by probit analysis for each virus: EEEV (23 PFU), VEEV (0.018 PFU), and WEEV (11 PFU). For VEEV, (?) indicates calculated challenge dose could not be determined due to plaque assay sensitivity limits.

To determine how sensitive hamsters are to aerosol exposure of EEEV, VEEV, and WEEV, the hamsters were exposed to a dose range of each virus and monitored for morbidity and mortality. The hamsters demonstrated comparable sensitivity to the encephalitic alphaviruses after aerosol exposure as seen in other rodent models [36,37,38,39,40]. The lethal dose 50 (LD50) was the lowest for hamsters exposed to VEEV (Figure 1B) with a dose of 3 logs lower virus resulting in the LD50 (0.018 PFU) compared to hamsters exposed to EEEV (Figure 1A) and WEEV (Figure 1C). Hamsters exposed to WEEV and EEEV had comparable doses resulting in LD50 (11 PFU vs. 23 PFU, respectively).

### 3.2. Clinical Signs in Hamsters following Aerosol Exposure to Encephalitic Alphaviruses

Hamsters infected with the encephalitic alphaviruses presented with limited clinical signs of disease such as limited weight loss (≤10% of starting weight) (Figure 1D–F), reduced grooming, dull/rough coat, lack of grooming/piloerection, ocular/nasal discharge, and hunched posture occurring 12–24 h before succumbing to infection. This is similar to what has been previously shown for other rodent models for EEEV and WEEV [38,39,41]; however, this is dramatically different from what has been observed in mice infected with VEEV, where mice exhibited prolonged clinical signs of disease including ruffled fur and dramatic weight loss starting as soon as 24 h post insfection [41,42,43].

**Figure 2:**
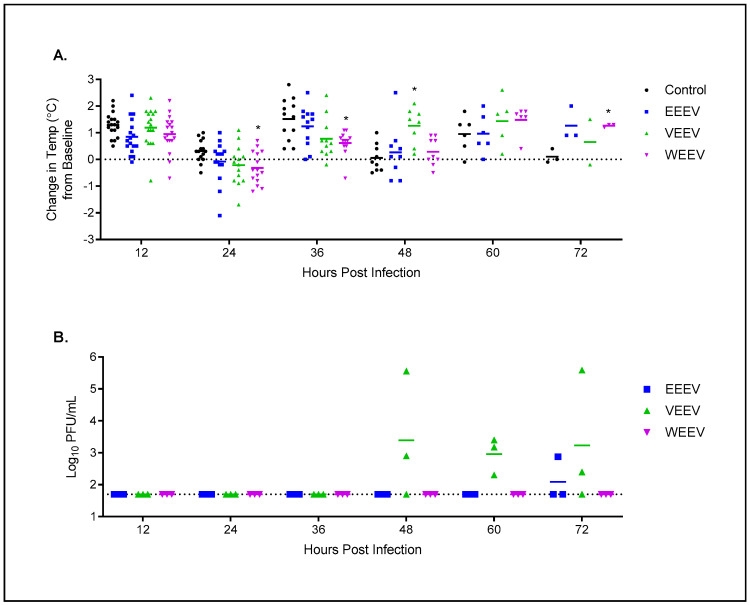
Temperature and viremia evaluation in hamsters after aerosol exposure to encephalitic alphaviruses. Eight- to ten-week-old hamsters of both sexes (*n* = 18 per group) were either mock-exposed or exposed to 100 LD50 via aerosol of EEEV, VEEV, or WEEV. Hamsters were implanted with IPTT temperature probe and temperature was obtained every 12 h for 72 h. (**A**) Change in temperature is based on baseline temperature before aerosol exposure. At every 12 h, three hamsters were euthanized for pathology. * = *p* ≤ 0.05. (**B**) At each time point *n* = 3 hamsters were euthanized and blood and tissues were harvested. Blood was used to determine viremia by a standard plaque assay. Dotted line represents the limit of detection for the plaque assay.

Hamsters were also monitored for alteration in temperature and temperature was monitored every 12 h for 72 h. As expected, the hamsters displayed classical diurnal temperature fluctuations (Figure 2A). Hamsters exposed to EEEV had no statistically significant changes in temperature compared to the control animals. Hamsters exposed to VEEV exhibited elevated temperature compared to the control animals at 48 hpi (*p* ≤ 0.05) while hamsters exposed to WEEV had significantly lower temperature at 24 and 36 hpi (*p* ≤ 0.05) and significantly higher temperature at 72 hpi (*p* ≤ 0.05).

Additionally, viremia in the blood was tested in hamsters that were sacrificed at pre-determined time points for the pathology (Figure 2B). Hamsters exposed to WEEV did not have detectable viremia and only one hamster exposed to EEEV had detectable viremia at 72 hpi. The lack of viremia in the hamsters exposed to EEEV is inconsistent with what is observed in mice [39,41,44] and NHPs [45,46,47]. However, the lack of viremia in hamsters exposed to WEEV is consistent with what is observed in the NHP model [13,46,47,48,49]. Viremia was detected in hamsters exposed to VEEV, but the timing is delayed compared to what is observed in both mice [41,50,51,52] and NHPs [45,47,53].

### 3.3. Pathology in Hamsters following Aerosol Exposure to Encephalitic Alphaviruses

Next, the pathological changes after aerosol exposure to the encephalitic alphaviruses were examined. Hamsters were exposed to a dose of 100 LD50 of virus and three hamsters were sacrificed every 12 h until 72 h (same animals from Figure 2). Pathological changes and immunohistochemistry for viral antigen were examined in various tissues including central nervous system (CNS) tissues, duodenum, and reproductive tissues (EEEV Table 1 and Table 2; VEEV Table 3 and Table 4; WEEV Table 5 and Table 6). Histological changes are summarized in Table 1, Table 3 and Table 5 for hamsters exposed to EEEV, VEEV, and WEEV, respectively, and immunohistochemical staining for viral antigen is summarized in Table 2, Table 4 and Table 6 for hamsters exposed to EEEV, VEEV, and WEEV, respectively.

**Figure 3:**
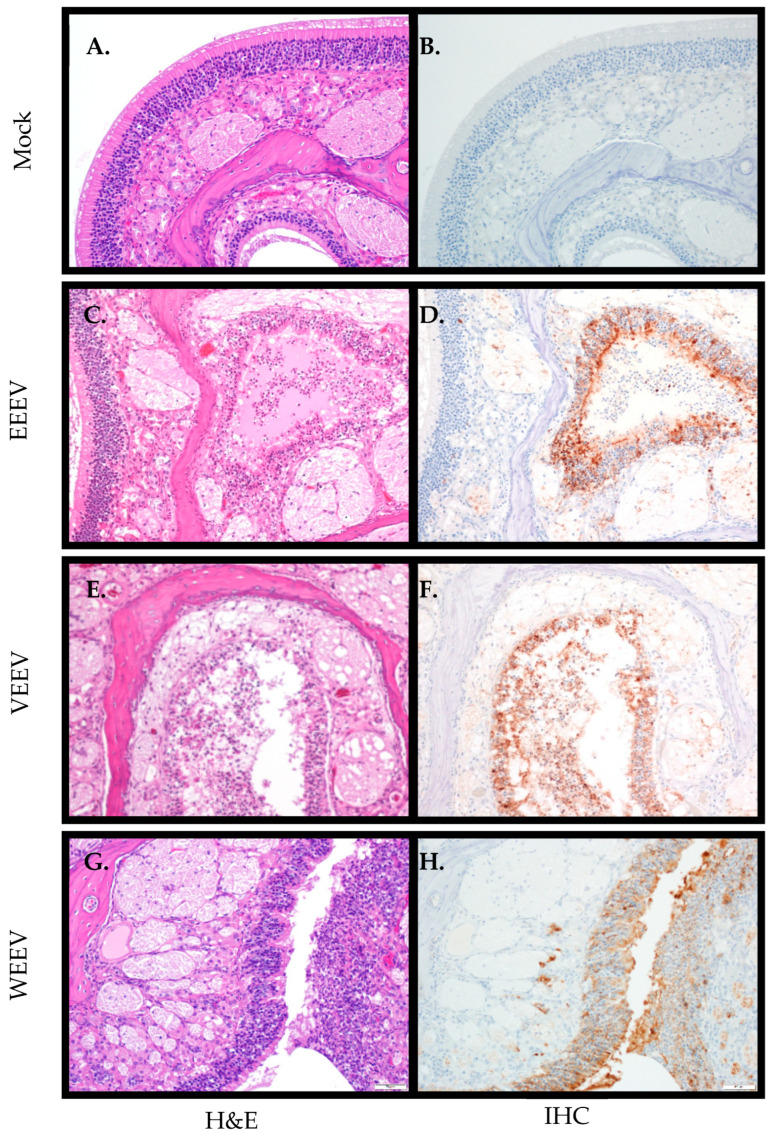
Histological and immunohistochemical changes in nasal turbinates after aerosol exposure to encephalitic alphaviruses. Eight- to ten-week-old hamsters of both sexes were either mock-exposed (**A**,**B**) or exposed to 100 LD50 via aerosol of EEEV (**C**,**D**), VEEV (**E**,**F**), or WEEV (**G**,**H**). Nasal turbinates were collected and stained with H&E and examined for histological changes (20× magnification; **A**,**C**,**E**,**G**) or immunohistochemistry for viral antigen (20× magnification; **B**,**D**,**F**,**H**). Mock-infected hamsters exhibited normal histology in the nasal turbinates (**A**) and no staining for alphavirus antigen (**B**). Hamsters exposed to virus demonstrated significant pathology in the nasal turbinates (**C**,**E**,**G**). Hamsters exposed to EEEV had significant necrosis in the nasal turbinates with sloughed olfactory epithelial cells and neutrophils and proteinaceous exudate in the lumen (**C**). Hamsters exposed to VEEV also had significant necrosis of olfactory epithelium with infiltrating neutrophils (**E**). Hamsters exposed to WEEV also had significant necrosis of olfactory epithelium and vacuolation of olfactory nerve (spongiosis) (**G**). Viral antigen was detected in the olfactory epithelium and nerves of hamsters exposed to EEEV, VEEV, and WEEV (**D**,**F**,**H**).

Nasal cavity/olfactory epithelium: After an aerosol exposure, one of the first sites of replication is the nasal cavity/olfactory epithelium. Both histological changes were evaluated along with immunohistochemistry for viral antigen in the nasal cavity. Hamsters exposed to EEEV did not begin to consistently demonstrate both histological changes and positive antigen staining until 72 hpi (Table 1 and Table 2). Only one animal at 24 hpi had mild pathology (Table 1) and antigen staining only in the nasal cavity (Table 2), and an additional animal at 60 hpi exhibited significant histological changes along with antigen staining. In the nasal turbinate, necrosis of olfactory epithelial was observed with sloughing of epithelial cells, infiltration of neutrophils, and proteinaceous exudate in lumen (Figure 3C). The olfactory epithelium had positive viral antigen staining, and there was weak scattered antigen staining of nerves (Figure 3D). Two of three animals exposed to VEEV had positive virus staining in the nasal cavity by 48 hpi with mild pathology, and by 60 hpi all animals had positive virus staining and pathological changes in the nasal cavity (Table 3 and Table 4). In the nasal cavity, there was necrosis of olfactory epithelium and vacuolation of olfactory nerve and spongiosis (Figure 3E) as well as positive antigen staining in the olfactory epithelium and nerves (Figure 3F). Interestingly, hamsters exposed to WEEV demonstrated the earliest consistent pathological changes and positive virus antigen staining in the nasal cavity at 24 hpi (Table 5 and Table 6). The olfactory epithelium was necrotic with infiltrating neutrophils (Figure 3G) and positive virus antigen staining in the olfactory epithelium and nerves (Figure 3H). Not unexpectedly, all three viruses were able to efficiently replicate in the nasal cavity resulting in histopathological changes, although the timing was different.

**Figure 4:**
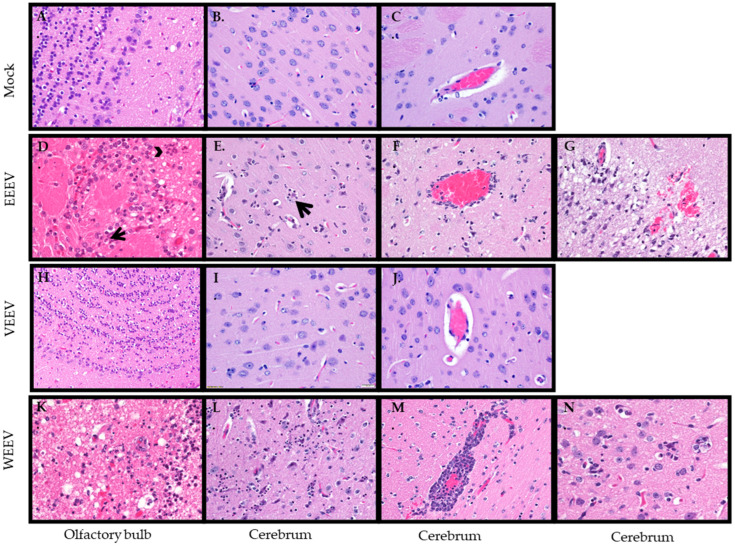
Histological changes in central nervous system after aerosol exposure to encephalitic alphaviruses. Eight- to ten-week-old hamsters of both sexes were either mock-exposed (**A**–**C**) or exposed to 100 LD50 via aerosol of EEEV (**D**–**G**), VEEV (**H**–**J**), or WEEV (**K**–**N**). Olfactory bulb (**A**,**D**,**H**,**K**) and cerebrum (**B**,**C**,**E**–**G**,**I**,**J**,**L**–**N**) were collected and stained with H & E and examined for histological changes (magnification 40×). Mock-exposed animals had normal histology in the olfactory bulb (**A**) and cerebellum (**B**–**C**) and around the blood vessel (**C**). In the olfactory bulb, hamsters exposed to EEEV had individual cell necrosis (arrow) with few neutrophils (arrowhead) and hemorrhaging (**D**), hamsters exposed to VEEV had rare necrotic/apoptotic-like cellular debris and mild, multifocal spongiosis and hemorrhaging (**H**), and hamsters exposed to WEEV had vacuolation of neuropil (spongiosis), scattered individual cell necrosis, and neutrophil infiltration and hemorrhaging (**K**). In the cerebrum, hamsters exposed to EEEV, had degenerate neurons surrounded by phagocytic cells (neuronophagia; arrow) (**E**), expansion of perivascular space with neutrophils and extension into adjacent neuropil (**F**), and vacuolation of neuropil (spongiosis), neuronal degradation, and necrosis with neutrophil infiltration with focal hemorrhage (**G**). Hamsters exposed to VEEV had normal histology in the cerebrum (**I**,**J**) even around blood vesicles (**J**). Hamsters exposed to WEEV had neuronal degeneration and necrosis with satellitosis in the cerebrum (**L**), along with perivascular spaces predominantly expanded by lymphocytes (**M**) with focal hemorrhaging (**M**,**N**) and degenerative neurons surrounded by phagocytic cells (neuronophagia) (**N**).

**Figure 5:**
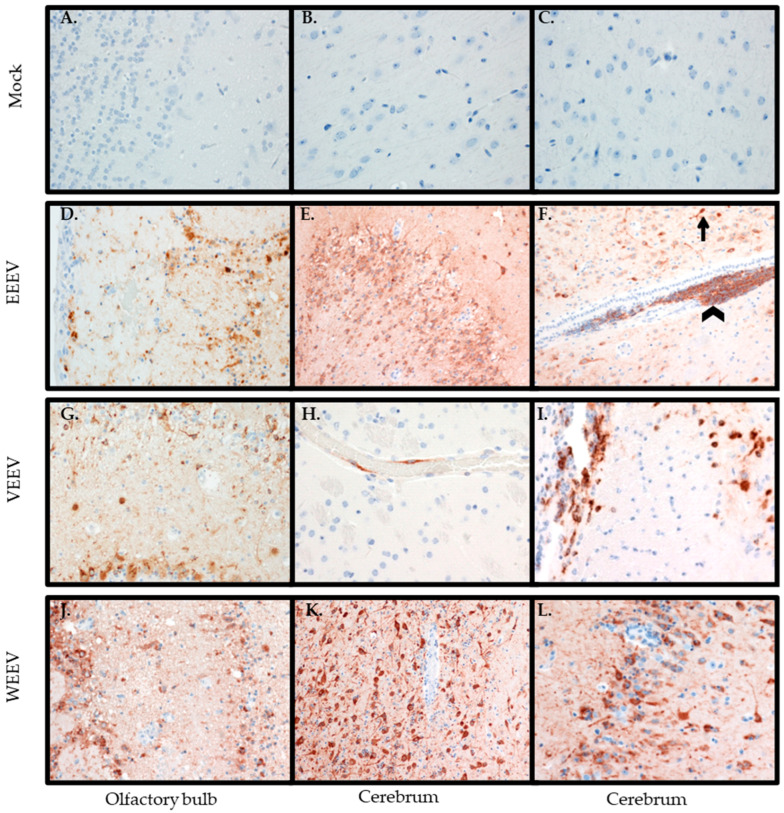
Immunohistochemical staining of central nervous system tissues for alphavirus antigen after aerosol exposure. Eight- to ten-week-old hamsters of both sexes were either mock-exposed (**A**–**C**) or exposed to 100 LD50 via aerosol of EEEV (**D**–**F**), VEEV (**G**–**I**), or WEEV (**J**–**L**). Olfactory bulb (**A**,**D**,**G**,**J**) and cerebrum (**B**,**C**,**E**,**F**,**H**,**I**,**K**,**L**) were collected and immunohistochemical stained for viral antigen (magnification 40×). Mock-exposed hamsters were negative for viral antigen in both the olfactory bulb (**A**) and cerebrum (**B**–**C**). In the olfactory bulb, EEEV (**D**), VEEV (**G**), and WEEV (**J**)-exposed hamsters had neurons that were positive for viral antigen, and EEEV and WEEV-exposed hamsters also had nerve fibers that were also positive for viral antigen. In the cerebrum, EEEV-exposed hamsters had neurons ((**E**,**F**) (arrow)) and glial cells ((**F**); arrowhead) positive for antigen. VEEV-exposed hamsters had endothelial cells (**H**) and neurons and glial cells (**I**) positive for viral antigen. WEEV-exposed hamsters also had neurons positive for viral antigen (**K**).

CNS: A hallmark of the encephalitic alphaviruses is replication within the CNS. Both histological changes and immunohistochemistry were evaluated for viral antigen within the CNS. Hamsters exposed to EEEV exhibited a delay in entry into the CNS with viral antigen staining not consistently detected until 72 hpi (Table 2). The olfactory bulb had individual cell necrosis with a few infiltrating neutrophils, mild spongiosis, and hemorrhage (Figure 4D) with positive viral antigen staining (Figure 5D). The cerebrum exhibited necrosis, degeneration of neurons with neuronophagia (Figure 4E), and expansion of perivascular space with infiltrating neutrophils and extension into adjacent neuropil (Figure 4F), vacuolation of neuropil (spongiosis), and focal hemorrhage (Figure 4G). Viral staining was mainly in the piriform cortex, dentate gyrus of the hippocampus, and thalamus of the cerebrum. Both neurons and glial cells stained positive for viral antigen; however, the ependymal cells appeared negative for virus (Figure 5E,F). Interestingly, EEEV viral antigen was detected simultaneously in the CNS and peripheral tissues (Table 2).

Hamsters exposed to VEEV had some viral antigen staining in the CNS at 48 hpi, but consistent staining did not occur in the CNS until 60 hpi (Table 4). Although there was viral antigen staining in the olfactory bulb (Figure 5G) and in the cerebrum in the endothelial cells (Figure 5H), neurons, and glial cells (Figure 5I), there was minimal pathology observed in the brain (Figure 4I,J). The olfactory bulb was the most affected, although still minimally, with rare necrotic/apoptotic-like cellular debris and mild, multifocal spongiosis observed (Figure 4H).

Hamsters exposed to WEEV first had viral antigen detected in the olfactory bulb at 36 hpi (Table 6) with minimal histological changes (Table 5), and as the infection progressed the amount of pathology caused by the virus increased (Table 5) along with the amount of antigen staining in the olfactory bulb (Table 6). The histological changes in the olfactory bulb include spongiosis with scattered individual cell necrosis and neutrophil infiltration, along with hemorrhaging (Figure 4K). Additionally, neurons in the olfactory bulb stained positive for viral antigen (Figure 5J). In the cerebrum, there was neuronal degeneration and necrosis with satellitosis (Figure 4L), degenerate neurons surrounded by phagocytic cells (neuronophagia) (Figure 4N). The perivascular spaces were predominantly expanded by lymphocytes (Figure 4M), and focal hemorrhaging was detected (Figure 4M,N). Viral antigen staining was mainly in the piriform cortex, hippocampus, and thalamus of the cerebrum (Figure 5K,L). Neurons in these areas were positive for viral antigen.

Although viral antigen staining was present in the CNS for each encephalitic alphavirus, there was a difference in timing of entry of the virus into the CNS and the amount of pathology induced by the different viruses. Of notable interest, was the lack of pathology in the CNS caused by VEEV, although there was viral antigen staining and the hamsters succumbed to infection.

**Figure 6:**
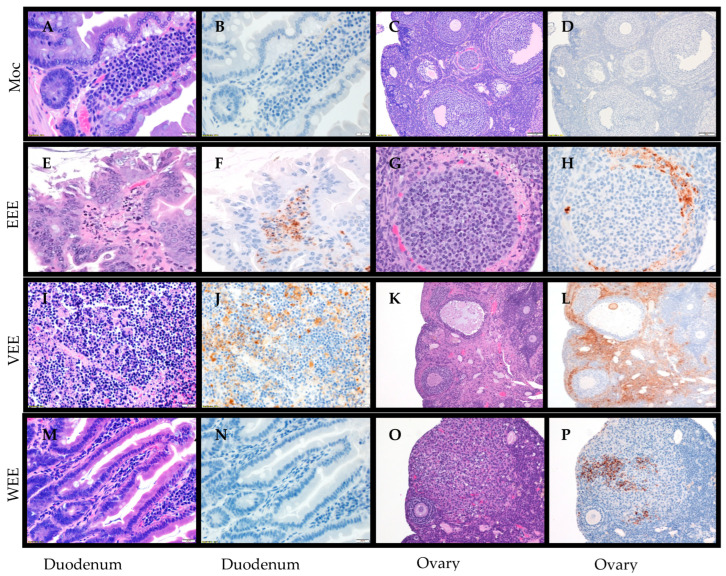
Histological and immunohistochemical changes in duodenum and ovaries after aerosol exposure to encephalitic alphaviruses. Eight- to ten-week-old hamsters of both sexes were either mock-exposed (**A**–**D**) or exposed to 100 LD50 via aerosol of EEEV (**E**–**H**), VEEV (**I**–**L**), or WEEV (**M**–**P**). The duodenum and ovaries were collected and stained with H & E and examined for histological changes (**A**,**C**,**E**,**G**,**I**,**K**,**M**,**O**) or immunohistochemistry for viral antigen (**B**,**D**,**F**,**H**,**J**,**L**,**N**,**P**). Mock-infected hamsters exhibited normal histology in the duodenum (**A**) and ovaries (**C**) and no staining for viral antigen (**B**,**D**). In the duodenum, hamsters exposed to EEEV had necrotic debris in the lamina propria (**E**) with antigen staining of necrotic cells (**F**). Hamsters exposed to VEEV exhibited necrosis of Peyer’s patch and mucosa with hemorrhaging (**I**) with antigen staining of macrophages, enterocytes, spindle cells, and endothelial cells in the lamia propria (**J**). Hamsters exposed to WEEV had no histological changes (**M**) or antigen staining (**N**) in the duodenum. In the ovary follicle, hamsters exposed to EEEV had necrosis of the thecal layer (**G**) with the thecal and necrotic cells positive for viral antigen (**H**). Hamsters exposed to VEEV also had necrosis in the ovaries (**K**) with viral antigen staining of endothelial cells, thecal cells, and necrotic cells (**L**). Hamsters exposed to WEEV had necrotic debris in the ovary follicle with a low number of infiltrating neutrophils (**O**), and the necrotic cells were positive for viral antigen (**P**).

Duodenum: It has been previously reported that hamsters infected with VEEV via SC had extensive replication, hemorrhage, and necrosis in the intestines [18,24,54]; therefore, it was desired to determine if hamsters exposed via the aerosol route exhibited a similar phenotype. Hamsters exposed to aerosolized EEEV demonstrated necrotic debris in the lamina propria (Figure 6E) with viral antigen staining in necrotic cells (Figure 6F). Additionally, VEEV-aerosol-exposed hamsters had viral antigen staining in the duodenum (Figure 6J) with the Peyer’s patch having necrosis with mucosa hemorrhage (Figure 6I). Interestingly, WEEV-aerosol-exposed animals appeared to have no histological changes (Figure 6M) and no viral antigen staining in the duodenum (Figure 6N). These findings support that the hamster model has a propensity for extensive necrosis and hemorrhage in the intestines after virus exposure independent of the route of exposure.

Reproductive organs: Since alphaviruses are capable of replicating in the reproductive organs [36,55,56,57], viral antigen and histology was examined in the reproductive organs after aerosol exposure. The time points alternated between males and females with males being used at 12, 36, and 60 hpi while females were used at 24, 48, and 72 hpi. Hamsters exposed to EEEV had necrosis of the thecal layer in the ovaries (Figure 6G) with viral antigen staining in the thecal and necrotic cells (Figure 6H). Additionally, necrosis was also observed in the uterus (Table 1). Hamsters exposed to VEEV also had necrosis in the ovary (Figure 6L) with viral antigen staining of the thecal layer and in necrotic cells. Additionally, there was endometrial necrosis of the uterus and rare spermatogonia in the testes observed (Table 3). Hamsters exposed to WEEV had ovaries with necrotic debris and neutrophilic inflammation (Figure 6O) with the necrotic cells staining positive for viral antigen (Figure 6P). Since each time point was alternating between males and females, it is possible that there would be more pathology in the testes had male hamsters been used at 72 hpi instead of female hamsters. All three viruses were able to replicate efficiently in the reproductive organs of the hamsters and cause extensive pathology.

Other tissues: Interestingly, while hamsters exposed to EEEV and VEEV had marked necrosis/apoptosis in the teeth (Table 1 and Table 3, respectively) with strong viral antigen staining (Table 2 and Table 4, respectively), hamsters exposed to WEEV had little or no pathology (Table 5) observed in the teeth and had inconsistently mild to moderate viral antigen staining (Table 6). Not unexpectedly, hamsters exposed to VEEV had the widest range of tropism for different cell type/tissues, especially lymphoid tissues and the pancreas (Table 3 and Table 4), with little to no viral antigen staining in lymphoid tissues and the pancreas in the hamsters exposed to EEEV (Table 1 and Table 2) and WEEV (Table 5 and Table 6).

## 4. Discussion

With the limited number of humans infected each year with the encephalitic alphaviruses, for human efficacy trials to be conducted, FDA licensure of vaccines and therapeutics would have to be conducted through the FDA animal rule. Potential products would first need to be tested in a small animal model for down-selection before being moved to the NHP model. Small animal models offer a rapid and reasonably inexpensive model for screening vaccines and therapeutics that can then be moved on to the more expensive, labor intensive NHP model, which limited facilities can accommodate. The FDA stipulates that the model must capitulate human diseases. The current small animal models are highly sensitive to the encephalitic alphaviruses and result in rapid mortality after exposure [16]. Especially for VEEV, this does not mimic human disease, because VEEV is rarely fatal in humans [4,15].

The hamster model was evaluated against aerosolized VEEV, EEEV, and WEEV to determine if this would be a good model for evaluating medical countermeasures against encephalitic alphaviruses. Like other small animal models tested [16] and previous work with hamsters infected either by the SC [17,18,19,20,21,22,23,24,25,26,27,28] or IP [12,18,22,29,30,31,32] route, the hamsters were highly sensitive to aerosol exposure to the encephalitic alphaviruses and succumbed to infection at low doses (Figure 1). The clinical signs of disease in the hamsters infected with aerosolized EEEV and WEEV were similar to what has been reported for other rodent models [38,39,41] with few clinical signs of infection before succumbing to infection. Surprisingly, hamsters exposed to aerosolized VEEV also showed very few clinical signs of disease before succumbing to infection, and this is vastly different from what has been reported for other rodent models [41,42,43]. This data started to suggest that the hamster model may not be a good model for studying aerosol exposure to the encephalitic alphaviruses.

An issue that has been observed with the hamsters challenged with VEEV via the SC route is they had extensive hemorrhage and necrosis of the intestinal wall [18,24,29,54] and succumbed to infection due to an endotoxin shock syndrome instead of encephalitis [19] making the SC route not the best model for studying VEEV. When histological changes and viral antigen staining were evaluated in various tissues, there was a concern about the extensive necrosis and hemorrhaging found in the intestines of animals infected with VEEV (Table 3 and Table 4; Figure 6) as this could lead to the mortality observed in these animals instead of mortality resulting from encephalitis as was observed after SC challenge [18,24,29,54]. To further support this premise for hamsters exposed to aerosolized VEEV, there was little pathology observed in the CNS of infected hamsters (Table 3; Figure 4) although there was viral antigen detected in the CNS (Table 3; Figure 5). Hamsters infected with SC VEEV have also demonstrated other physiological issues such as infection of the pancreas and altered insulin [25,58] that has not been observed in humans [59] or NHPs [60]. It was also observed that hamsters infected with VEEV via aerosol had a correlation between levels of viral antigen staining (Table 4) and histological changes (Table 3) in the pancreas. Similar to the VEEV-infected hamsters, the EEEV-infected hamsters (Table 1 and Table 2; Figure 6) had extensive necrosis and hemorrhaging in the intestines. The necrosis of the intestines along with the short survival time of the hamsters (Figure 1) make them an undesirable model for therapeutic studies, especially since mice [39,41,46,61] and NHPs [45,46,47,62,63] have a longer therapeutic window for treatment. The rapid onset of WEEV disease in the hamsters, similar to EEEV, results in a very short therapeutic window for medical countermeasure development that is not observed in the NHP model [13,46,47]. Additionally, WEEV is highly lethal in the hamsters at low doses, while most studies with NHPs are with high doses with variable lethality [8,13,46,47]. Additionally, all three encephalitic alphaviruses caused extensive histological changes and viral antigen staining in the reproductive organs (Table 1, Table 2, Table 3, Table 4, Table 5 and Table 6; Figure 6) that could alter outcomes of infection. Ideally, the same animal model would be used to evaluate therapeutics and vaccines with the encephalitic alphaviruses since many of the encephalitic alphavirus vaccines are being developed as a tri-valent formulation and therapeutics are being evaluated for broad anti-alphavirus activity. The results from this aerosolized alphaviral pathogenesis study provide further evidence that the hamster model is not an appropriate animal model for testing medical countermeasures against encephalitic alphaviruses.

## Figures and Tables

**Table 1:** Histological changes in hamsters after aerosol exposure to EEEV.

Tissue Examined	Lesions	12 h			24 h			36 h			48 h			60 h			72 h		
		1	2	3	1	2	3	1	2	3	1	2	3	1	2	3	1	2	3
Nasal cavity	Inflammation	−	−	−	−	−	1, f	−	−	−	−	−	−	−	2, mf	−	3, mf	−	−
	Necrosis/apoptosis	−	−	−	−	−	−	−	−	−	−	−	−	−	+/−	−	+	+	+
Tooth	Necrosis/apoptosis	−	−	−	−	−	−	−	−	−	−	−	−	−	+/−	−	+	+	+
Brain, olfactory bulb	Inflammation	−	−	−	−	−	−	−	−	−	−	−	−	−	−	−	−	−	2, mf
	Necrosis/apoptosis	−	−	−	−	−	−	−	−	−	−	−	−	−	+/−	−	+	+/−	+
	Spongiosis	−	−	−	−	−	−	−	−	−	−	−	−	−	−	−	+	+	+
	Hemorrhage	−	−	−	−	−	−	−	−	−	−	−	−	−	−	−	+	+	+
Brain, cerebrum	Inflammation	−	−	−	−	−	−	−	−	−	−	−	−	−	1, mf	−	1, mf	1, mf	2, mf
	Necrosis/apoptosis	−	−	−	−	−	−	−	−	−	−	−	−	−	+/−	−	+	+	+
	Neuronophagia	−	−	−	−	−	−	−	−	−	−	−	−	−	−	−	−	+	+
	Hemorrhage	−	−	−	−	−	−	−	−	−	−	−	−	−	−	−	+	+	+
	Gliosis/satellitosis	−	−	−	−	−	−	−	−	−	−	−	−	−	−	−	+/−	+	+
Vomeronasal organ	Necrosis/apoptosis	−	−	−	−	−	−	−	−	−	−	−	−	−	+/−	−	−	−	−
Lymph node	Inflammation	−	−	−	−	−	−	−	−	−	−	−	−	−	2, mf	−	2–3, mf	2–3, mf	3, mf
	Necrosis	−	−	−	−	−	−	−	−	−	−	−	−	−	+, sub	−	+, sub	+, mes	−
	Lymphoid depletion	−	−	−	−	−	−	−	−	−	−	−	−	−		−	−	−	−
	Lymphocytolysis	−	−	−	−	−	−	−	−	−	−	−	−	−	+/−	−	+	+	+
Pancreas	Inflammation	−	−	−	−	−	−	−	−	−	−	−	−	−	−	−	−	−	−
	Necrosis	−	−	−	−	−	−	−	−	−	−	−	−	−	−	−	−	−	−
	Lymphoid depletion				−	−	−	−	−	−	−	−	−	−	−	−	−	−	−
	Lymphocytolysis	−	−	−	−	−	−	−	−	−	−	−	−	−	+/−	−	+/−	+/−	+/−
GALT/Peyer’s patches	Necrosis/apoptosis	−	−	−	−	−	−	−	−	−	−	−	−	−		−	−	−	−
Reproductive tract	Inflammation	−	−	−	−	−	−	−	−	−	−	−	−	−	−	−	−	−	−
	Necrosis/apoptosis	−	−	−	−	−	−	−	−	−	−	−	−	−	−	−	+	+	+
Lung	Inflammation	−	−	−	−	−	−	−	−	−	−	−	−	−	1, mf	−	1, mf	1, mf	1, mf
	Necrosis/apoptosis	−	−	−	−	−		−	−	−	−	−	−	−		−	−	−	−
Adrenal gland	Inflammation	−	−	−	−	−	−	−	−	−	−	−	−	−	3, mf	−	1, mf	1, mf	2, mf
	Necrosis/apoptosis	−	−	−	−	−	−	−	−	−	−	−	−	−	+	−	+	+	+
Pituitary gland	Inflammation	−	−	−	−	−	−	−	−	−	−	−	−	−	−	−	−	−	−
	Necrosis/apoptosis				−	−	−	−	−	−	−	−	−	−	−	−	−	−	−
Small intestine	Necrosis/apoptosis	−	−	−	−	−	−	−	−	−	−	−	−	−	−	−	−	+/−	−
Stomach	Inflammation	−	−	−	−	−	−	−	−	−	−	−	−	−	−	−	−	−	−
	Necrosis/apoptosis	−	−	−	−	−	−	−	−	−	−	−	−	−	−	−	−	−	−
Colon	Necrosis/apoptosis	−	−	−	−	−	−	−	−	−	−	−	−	−	−	−	−	−	−
Liver	Inflammation	−	−	−	−	−	2, d	−	−	−	−	−	−	−	−	−	−	−	−
	Necrosis/apoptosis	−	−	−	−	−	+/−	−	−	−	−	−	−	−	−	−	−	−	−
Haired skin	Necrosis/apoptosis	−	−	−	−	−	−	−	−	−	−	−	−	−	−	−	−	−	−
Thymus	Lymphocytolysis	−	−	−	−	−	−	−	−	−	−	−	−	−	−	−	−	−	−
Bone marrow	Necrosis	−	−	−	−	−	−	−	−	−	−	−	−	−	−	−	−	−	−
Pancreas	Necrosis/apoptosis	−	−	−	−	−	−	−	−	−	−	−	−	−	−	−	−	−	−

A score of 1–5 indicates the severity of inflammation present in the examined tissue: 1 (minimal); 2 (mild); 3 (moderate); 4 (marked); 5 (severe). +, +/−, or—indicates if the entity was present and easily recognized (+), variably present throughout the tissue (+/−), or not detected histologically (−). Distribution: f = focal; mf = multifocal; d = diffuse. Location: Lymph node, sub = submandibular LN; mes = mesenteric LN.

**Table 2:** Immunohistochemical staining for EEEV antigen in hamsters after aerosol exposure to EEEV.

Tissue Examined	12 h			24 h			36 h			48 h			60 h			72 h		
	1	2	3	1	2	3	1	2	3	1	2	3	1	2	3	1	2	3
Nasal cavity	−	−	−	−	−	2	−	−	−	−	−	−	−	1–2	−	4	2	2
Tooth	−	−	−	−	−	−	−	−	−	−	−	−	−	3	−	3	3	3
Brain, olfactory bulb	−	−	−	−	−	−	−	−	−	−	−	−	−	3	−	4	4	4
Brain, cerebrum	−	−	−	−	−	−	−	−	−	−	−	−	−	4	−	4	4	4
Brain, brainstem	−	−	−	−	−	−	−	−	−	−	−	−	−	1	−	3	4	3
Brain, cerebellum	−	−	−	−	−	−	−	−	−	−	−	−	−	−	−	1	1	rare
Vomeronasal organ	−	−	−	−	−	−	−	−	−	−	−	−	−	1	−	−	−	1
Lymph node, axillary	−	−	−	−	−	−	−	−	−	−	−	−	−	1	−	−	−	1
Lymph node, mesenteric	−	−	−	−	−	−	−	−	−	−	−	−	−	1	−	1	1	1
Lymph node, popliteal	−	−	−	−	−	−	−	−	−	−	−	−	−	1	−	1	2	1
Lymph node, submandibular	−	−	−	−	−	−	−	−	−	−	−	−	−	2	−	2	tnp	1
Lymph node, inguinal	−	−	−	−	−	−	−	−	−	−	−	−	−	1	−	rare	1	1
Spleen	−	−	−				−	−	−	−	−	−	−	−	−	−	−	−
Peyer’s patches (GALT)	−	−	−	−	−	−	−	−	−	−	−	−	−	−	−	−	−	−
Bone marrow	−	−	−	−	−	−	−	−	−	−	−	−	−	−	−	−	−	−
Lung	−	−	−	−	−	−	−	−	−	−	−	−	−	1	−	1	1	1
Thymus	−	−	−	−	−	−	−	−	−	−	−	−	−	−	−	−	−	−
Pancreas	−	−	−				−	−	−	−	−	−	−	−	−	−	−	−
Liver	−	−	−	−	−	−	−	−	−	−	−	−	−	−	−	−	−	−
Small intestine	−	−	−	−	−	−	−	−	−	−	−	−	−	−	−	−	1	−
Stomach	−	−	−	−	−	−	−	−	−	−	−	−	−	−	−	−	−	−
Adrenal gland	−	−	−	−	−	−	−	−	−	−	−	−	−	3	−	2	3	3
Reproductive tract	−	−	−	−	−	−	−	−	−	−	−	−	−	−	−	3	4	4
Haired skin	−	−	−	−	−	−	−	−	−	−	−	−	−	−	−	−	2	−
Pituitary gland	−	−	−	−	−	−	−	−	−	−	−	−	−	−	−	−	−	−
Kidney	−	−	−	−	−	−	−	−	−	−	−	−	−	−	−	−	−	−
Large intestine	−	−	−	−	−	−	−	−	−	−	−	−	−	−	−	−	−	−
Heart	−	−	−	−	−	−	−	−	−	−	−	−	−	−	−	−	−	−
Thyroid gland	−	−	−	−	−	−	−	−	−	−	−	−	−	−	−	−	−	−
Pituitary gland	−	−	−	−	−	−	−	−	−	−	−	−	−	2	−	2	2	3

Intensity of staining was graded on the following scale: 1 = 1–10 cells/hpf; 2 = 11–20 cells/hpf; 3 = 21–40 cells/hpf; 4 = >40 cells/hpf. tnp = tissue not present; hpf=high-powered field.

**Table 3:** Histological changes in hamsters after aerosol exposure to VEEV.

Tissue Examined	Lesions	12 h			24 h			36 h			48 h			60 h			72 h		
		1	2	3	1	2	3	1	2	3	1	2	3	1	2	3	1	2	3
Nasal cavity	Inflammation	−	−	−	−	−	−	−	−	−	−	−	−	−	−	−	−	1, mf	−
	Necrosis/apoptosis	−	−	−	−	−	−	−	−	−	−	−	+	+	+	+	+	+	+
Tooth	Necrosis/apoptosis	−	−	−	−	−	−	−	−	−	+/−	−	+	+	+	+	+	+	+
Brain, olfactory bulb	Inflammation	−	−	−	−	−	−	−	−	−	−	−	−	−	−	−	−	−	−
	Necrosis/apoptosis	−	−	−	−	−	−	−	−	−	−	−	+/−	−	−	−	+/−	+/−	+/−
	Spongiosis	−	−	−	−	−	−	−	−	−	−	−	+/−	−	−	−	+	+	+
	Hemorrhage	−	−	−	−	−	−	−	−	−	−	−	−	−	−	−	−	−	−
Brain, cerebrum	Inflammation	−	−	−	−	−	−	−	−	−	−	−	−	−	−	−	−	−	−
	Necrosis/apoptosis	−	−	−	−	−	−	−	−	−	−	−	−	−	−	−	+/−	−	−
	Neuronophagia	−	−	−	−	−	−	−	−	−	−	−	−	−	−	−	−	−	−
	Hemorrhage	−	−	−	−	−	−	−	−	−	−	−	−	−	−	−	−	−	−
	Gliosis/satellitosis	−	−	−	−	−	−	−	−	−	−	−	−	−	−	−	−	−	−
Vomeronasal organ	Necrosis/apoptosis	−	−	−	−	−	−	−	−	−	−	−	−	−	−	+	+	+	+
Lymph node	Inflammation	−	−	−	−	−	−	1–2, f	−	−	3,mf	2, f	2, mf	2, mf	2, mf	2, mf	2, mf	2, mf	2, mf
	Necrosis	−	−	−	−	−	−	−	−	−	+	−	+	+	+	+	+	+	+
	Lymphoid depletion	−	−	−	−	−	−	−	−	−	+	−	+	+	+	+	+	+	+
	Lymphocytolysis	−	−	−	−	−	−	−	−	−	−	−	−	−	−	−	−	−	−
Spleen (white pulp)	Inflammation	−	−	−	−	−	−	−	−	1, f	3, mf	3, mf	3, d	2, mf	2, mf	2, mf	3, d	3, d	3, d
	Necrosis	−	−	−	−	−	−	−	−	−	+	−	+	+	+	+	+	+	+
	Lymphoid depletion	−	−	−	−	−	−	−	−	−	−	+	−	−	−	−	+	−	+
	Lymphocytolysis	−	−	−	−	−	−	−	−	−	−	−	−	−	−	−	−	−	−
GALT/Peyer’s patches	Necrosis/apoptosis	−	−	−	−	−	−	−	−	−	−	−	+	+	+	+	+	−	+
Reproductive tract	Inflammation	−	−	−	−	−	−	−	−	−	−	2,f	−	−	−	−	−	−	−
	Necrosis/apoptosis	−	−	−	−	−	−	−	−	−	+	+	+	−	−	−	+	+	+
Lung	Inflammation	−	−	−	−	−	−	−	1, f	−	1, mf	−	1, d	1, mf	1, mf	1, mf	2, mf	−	2, mf
	Necrosis/apoptosis	−	−	−	−	−	−	−	−	−	+/−	−	−	−	+/−	−	+/−	−	+/−
Adrenal gland	Inflammation	−	−	−	−	−	−	−	−	−	−	−	−	−	−	−	−	−	−
	Necrosis/apoptosis	−	−	−	−	−	−	−	−	−	+/−	−	+/−	−	−	−	+	+/−	+
Pituitary gland	Inflammation	−	−	−	−	−	−	−	−	−	−	−	−	−	−	−	−	−	−
	Necrosis/apoptosis	−	−	−	−	−	−	−	−	−	−	−	−	−	−	−	+/−	−	+/−
Small intestine	Necrosis/apoptosis	−	−	−	−	−	−	−	−	−	+	−	+/−	−	−	−	+	+/−	+/−
Stomach	Inflammation	−	−	−	−	−	−	−		−	−	−	−	−	−	−	−	−	−
	Necrosis/apoptosis	−	−	−	−	−	−	−	−	−	+/−	−	+/−	−	−	−	+	+/−	+/−
Colon	Necrosis/apoptosis	−	−	−	−	−	−	−	−	−	−	−	−	−	−	−	+/−	−	+/−
Liver	Inflammation	−	−	−	−	−	−	−	−	−	1, f	−	1, f	−	−	−	−	−	−
	Necrosis/apoptosis	−	−	−	−	−	−	−	−	−	+/−	−	−	−	−	−	+/−	−	+/−
Haired skin	Necrosis/apoptosis	−	−	−	−	−	−	−	−	−	+/−	−	+/−	−	−	−	−	−	−
Thymus	Lymphocytolysis	−	−	−	−	−	−	−	−	−	+/−	−	+/−	−	−	−	−	−	+
Bone marrow	Necrosis	−	−	−	−	−	−	−	−	−	−	−	+	−	+	+	+	+	+
Pancreas	Necrosis/apoptosis	−	−	−	−	−	−	−	−	−	−	−	−	−	−	−	+	−	+/−

A score of 1–5 indicates the severity of inflammation present in the examined tissue: 1 (minimal); 2 (mild); 3 (moderate); 4 (marked); 5 (severe). +, +/−, or—indicates if the entity was present and easily recognized (+), variably present throughout the tissue (+/−), or not detected histologically (−). Distribution: f = focal; mf = multifocal; d = diffuse.

**Table 4:** Immunohistochemical staining for VEEV antigen in hamsters after aerosol exposure to VEEV.

Tissue Examined	12 h			24 h			36 h			48 h			60 h			72 h		
	1	2	3	1	2	3	1	2	3	1	2	3	1	2	3	1	2	3
Nasal cavity	−	−	−	−	−	−	−	−	−	3	−	4	4	4	4	4	4	4
Tooth	−	−	−	−	−	−	−	−	−	2	1	2	4	3	3	2	2	2
Brain, olfactory bulb	−	−	−	−	−	−	−	−	−	1	−	3	3	3	3	3	3	3
Brain, cerebrum	−	−	−	−	−	−	−	−	−	−	−	2	2	1	2	3	2	2
Brain, brainstem	−	−	−	−	−	−	−	−	−	−	−	−	−	−	1	2	−	−
Brain, cerebellum	−	−	−	−	−	−	−	−	−	−	−	−	rare	−	1	1	−	1
Vomeronasal organ	−	−	−	−	−	−	−	−	−	−	−	2	1	1	2	2	2	2
Lymph node, axillary	−	−	−	−	−	−	1	−	1	3	2	3	tnp	2	3	4	3	3
Lymph node, mesenteric	−	−	−	−	−	−	1	−	1	2	2	3	3	3	2	2	3	2
Lymph node, popliteal	−	−	−	−	−	−	1	−	−	1	rare	3	tnp	2	3	3	3	3
Lymph node, submandibular	−	−	−	−	−	−	−	−	−	3	1	3	2	3	tnp	tnp	3	3
Lymph node, inguinal	−	−	−	−	−	−	−	−	−	3	1	3	3	3	3	3	3	3
Spleen	−	−	−	−	−	−	rare	−	1	2	2	2	2	2	2	3	3	3
Peyer’s patches (GALT)	−	−	−	−	−	−	−	−	−	−	1	2	2	2	2	3	−	2
Bone marrow	−	−	−	−	−	−	1	−	1	2	2	2	2	1	2	2	1	2
Lung	−	−	−	−	−	−	−	1	−	1	−	2	1	1	1	2	1	2
Thymus	−	−	−	−	−	−	−	−	−	1	−	1	1	1	tnp	1	rare	2
Pancreas	−	−	−	−	−	−	−	−	−	1	−	1	1	1	1	3	rare	1
Liver	−	−	−	−	−	−	−	−	−	1	rare	1	1	1	1	2	1	2
Small intestine	−	−	−	−	−	−	−	−	−	1	−	1	1	1	1	2	1	2
Stomach	−	−	−	−	−	−	−	−	−	1	−	1	−	−	1	1	1	1
Adrenal gland	−	−	−	−	−	−	−	−	−	1	1	1	−	−	1	2	rare	2
Reproductive tract	−	−	−	−	−	−	−	−	−	2	2	2	1	1	1	3	1	2
Haired skin	−	−	−	−	−	−	−	−	−	1	−	1	−	1	1	−	−	1
Pituitary gland	−	−	−	−	−	−	−	−	−	1	−	2	−	1	−	2	−	−
Kidney	−	−	−	−	−	−	−	−	−	−	−	rare	−	rare	rare	rare	rare	rare
Large intestine	−	−	−	−	−	−	−	−	−	−	−	−	−	−	−	−	−	−
Heart	−	−	−	−	−	−	−	−	−	−	−	−	−	−	−	rare	−	1
Thyroid gland	−	−	−	−	−	−	−	−	−	−	−	−	−	−	−	rare	−	−
Pituitary gland	−	−	−	−	−	−	−	−	−	1	−	2	−	1	−	2	−	−

Intensity of staining was graded on the following scale: 1 = 1–10 cells/hpf; 2 = 11–20 cells/hpf; 3 = 21–40 cells/hpf; 4 = >40 cells/hpf. tnp = tissue not present; hpf=high-powered field.

**Table 5:** Histological changes in hamsters after aerosol exposure to WEEV.

Tissue Examined	Lesions	12 h			24 h			36 h			48 h			60 h			72 h		
		1	2	3	1	2	3	1	2	3	1	2	3	1	2	3	1	2	3
Nasal cavity	Inflammation	−	−	−	1, f	1, f	1, f	2, mf	3, mf	3, mf	3, mf	3, mf	3, mf	4, mf	4, mf	4, mf	3, mf	4, mf	4, mf
	Necrosis/apoptosis	−	−	−	+/−	−	+/−	+	+	+	+	+	+	+	+	+	+	+	+
Tooth	Necrosis/apoptosis	−	−	−	−	−	−	−	−	−	−	−	−	−	−	+	−	−	−
Brain, olfactory bulb	Inflammation	−	−	−	−	−	−	−	−	−	−	−	−	−	2, mf	2, mf	2, mf	2, mf	2, mf
	Necrosis/apoptosis	−	−	−	−	−	−	−	−	−	−	−	+/−	+/−	+	+	+	+	+
	Spongiosis	−	−	−	−	−	−	+/−	−	−	+/−	−	+/−	+/−	−	−	+	+	+
	Hemorrhage	−	−	−	−	−	−	−	−	−	−	−	−	−	−	+	+	+	+
Brain, cerebrum	Inflammation	−	−	−	−	−	−	−	−	−	−	−	−	2, mf	2, mf	2, mf	3, mf	2, mf	2, mf
	Necrosis/apoptosis	−	−	−	−	−	−	−	−	−	−	−	−	−	+/−	+/−	+	+	+
	Neuronophagia	−	−	−	−	−	−	−	−	−	−	−	−	−	−	−	+	+	+
	Hemorrhage	−	−	−	−	−	−	−	−	−	−	−	−	−	−	−	+	−	+
	Gliosis/satellitosis	−	−	−	−	−	−	−	−	−	−	−	−	−	−	−	−	−	−
Vomeronasal organ	Necrosis/apoptosis	−	−	−	−	−	−	−	−	−	+	+	+	+	+	+	+	+	+
Lymph node	Inflammation	−	−	−	−	−	−	−	−	−	−	−	−	−	−	−	−	−	−
	Necrosis	−	−	−	−	−	−	−	−	−	−	−	−	−	−	−	−	−	−
	Lymphoid depletion	−	−	−	−	−	−	−	−	−	−	−	−	−	−	−	−	−	−
	Lymphocytolysis	−	−	−	−	−	−	−	−	+/−	+/−	+/−	+/−	+	+	+	+	+	−
Spleen (white pulp)	Inflammation	−	−	−	−	−	−	−	−	−	−	−	−	−	−	−	−	−	−
	Necrosis	−	−	−	−	−	−	−	−	−	−	−	−	−	−	−	−	−	−
	Lymphoid depletion	−	−	−	−	−	−	−	−	−	−	−	−	−	−	−	−	−	−
	Lymphocytolysis	−	−	−	−	−	−	−	−	−	−	−	−	+/−	+/−	+/−	+/−	−	−
GALT/Peyer’s patches	Necrosis/apoptosis	−	−	−	−	−	−	−	−	−	−	−	−	−	−	−	−	−	−
Reproductive tract	Inflammation	−	−	−	−	−	−	−	−	−	−	−	−	−	−	−	2, mf	2, mf	2, f
	Necrosis/apoptosis	−	−	−	−	−	−	−	−	−	−	+/−	−	−	−	−	+	+	+/−
Lung	Inflammation	−	−	−	−	−	−	−	−	−	−	−	−	−	−	−	−	−	−
	Necrosis/apoptosis	−	−	−	−	−	−	−	−	−	−	−	−	−	−	−	−	−	−
Adrenal gland	Inflammation	−	−	−	−	−	−	−	−	−	−	−	−	−	−	−	−	−	−
	Necrosis/apoptosis	−	−	−	−	−	−	−	−	−	−	−	−	−	−	−	−	−	−
Pituitary gland	Inflammation	−	−	−	−	−	−	−	−	−	−	−	−	1, f	−	−	1, f	1, mf	−
	Necrosis/apoptosis	−	−	−	−	−	−	−	−	−	−	−	−	+/−	−	−	+/−	+	−
Small intestine	Necrosis/apoptosis	−	−	−	−	−	−	−	−	−				−	−	−	−	−	−
Stomach	Inflammation	−	−	−	−	−	−	−	−	−	−	−	−	−	−	−	−	2, f	−
	Necrosis/apoptosis	−	−	−	−	−	−	−	−	−	−	−	−	−	−	−	−	−	−
Colon	Necrosis/apoptosis	−	−	−	−	−	−	−	−	−	−	−	−	−	−	−	−	−	−
Liver	Inflammation	−	−	−	−	−	−	−	−	−	−	−	−	−	−	−	−	−	−
	Necrosis/apoptosis	−	−	−	−	−	−	−	−	−	−	−	−	−	−	−	−	−	−
Haired skin	Necrosis/apoptosis	−	−	−	−	−	−	−	−	−	−	−	−	−	−	−	−	−	−
Thymus	Lymphocytolysis	−	−	−	−	−	−	−	−	−	−	−	−	−	−	−	−	−	−
Bone marrow	Necrosis	−	−	−	−	−	−	−	−	−	−	−	−	−	−	−	−	−	−
Pancreas	Necrosis/apoptosis	−	−	−	−	−	−	−	−	−	−	−	−	−	−	−	−	−	−

A score of 1–5 indicates the severity of inflammation present in the examined tissue: 1 (minimal); 2 (mild); 3 (moderate); 4 (marked); 5 (severe). +, +/−, or—indicates if the entity was present and easily recognized (+), variably present throughout the tissue (+/−), or not detected histologically (−). Distribution: f = focal; mf = multifocal; d = diffuse.

**Table 6:** Immunohistochemical staining for EEEV antigen in hamsters after aerosol exposure to WEEV.

Tissue Examined	12 h			24 h			36 h			48 h			60 h			72 h		
	1	2	3	1	2	3	1	2	3	1	2	3	1	2	3	1	2	3
Nasal cavity	−	−	−	2	3	3	3	3	3	3	−	3	3	4	4	3	3	3
Tooth	−	−	−	−	−	−	2	1	−	−	−	−	−	−	2	−	1	−
Brain, olfactory bulb	−	−	−	−	1	−	2	2	2	2	−	3	3	4	4	4	4	4
Brain, cerebrum	−	−	−	−	−	−	−	−	−	2	2	3	3	3	3	4	3	4
Brain, brainstem	−	−	−	−	−	−	−	−	−	−	−	−	1	−	−	−	−	−
Brain, cerebellum	−	−	−	−	−	−	−	−	−	−	−	−	−	−	−	−	−	−
Vomeronasal organ	−	−	−	−	−	−	−	−	−	1	−	1	2	2	1	1	2	2
Lymph node, axillary	−	−	−	−	−	−	−	−	−	−	−	−	−	−	−	−	−	−
Lymph node, mesenteric	−	−	−	−	−	−	−	−	−	−	−	−	−	−	−	−	−	−
Lymph node, popliteal	−	−	−	−	−	−	−	−	−	−	−	−	−	−	−	−	−	−
Lymph node, submandibular	−	−	−	−	−	−	−	−	−	−	−	−	tnp	−	−	−	−	−
Lymph node, inguinal	−	−	−	−	−	−	−	−	−	−	−	−	−	−	−	−	−	−
Spleen	−	−	−	−	−	−	−	−	−	−	−	−	−	−	−	−	−	−
Peyer’s patches (GALT)	−	−	−	−	−	−	−	−	−	−	−	−	−	−	−	−	−	−
Bone marrow	−	−	−	−	−	−	−	−	−	−	−	−	−	−	−	−	−	−
Lung	−	−	−	−	−	−	−	−	−	−	−	−	1	1	−	−	−	−
Thymus	−	−	−	−	−	−	−	−	−	−	−	−	−	−	−	−	−	−
Pancreas	−	−	−	−	−	−	−	−	−	−	−	−	−	−	−	−	−	−
Liver	−	−	−	−	−	−	−	−	−	−	−	−	−	−	−	−	−	−
Small intestine	−	−	−	−	−	−	−	−	−	−	−	−	−	−	−	−	−	−
Stomach	−	−	−	−	−	−	−	−	−	−	−	−	−	−	−	−	1	−
Adrenal gland	−	−	−	−	−	−	−	−	−	−	−	−	−	−	−	−	−	−
Reproductive tract	−	−	−	−	−	−	−	−	−	−	1	−	−	−	−	1	2	2
Haired skin	−	−	−	−	−	−	−	−	−	−	−	−	−	−	−	−	−	−
Pituitary gland	−	−	−	−	−	−	−	−	−	−	1	−	1	1	−	1	2	1
Kidney	−	−	−	−	−	−	−	−	−	−	−	−	−	−	−	−	−	−
Large intestine	−	−	−	−	−	−	−	−	−	−	−	−	−	−	−	−	−	−
Heart	−	−	−	−	−	−	−	−	−	−	−	−	−	−	−	−	−	−
Thyroid gland	−	−	−	−	−	−	−	−	−	−	−	−	−	−	−	−	−	−
Pituitary gland	−	−	−	−	−	−	−	−	−	−	−	−	−	−	−	−	−	−

Intensity of staining was graded on the following scale: 1 = 1–10 cells/hpf; 2 = 11–20 cells/hpf; 3 = 21–40 cells/hpf; 4 = >40 cells/hpf. tnp = tissue not present; hpf=high-powered field.

## Data Availability

The data presented in this study are provided within the article and available upon request from the corresponding author.
